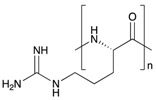# Layer-By-Layer Assemblies of Biopolymers: Build-Up, Mechanical Stability and Molecular Dynamics

**DOI:** 10.3390/polym12091949

**Published:** 2020-08-28

**Authors:** Jack Campbell, Anna S. Vikulina

**Affiliations:** 1 School of Science and Technology, Nottingham Trent University, Clifton Lane, Nottingham NG11 8NS, UK; jack.campbell@ntu.ac.uk; 2 Fraunhofer Institute for Cell Therapy and Immunology, Branch Bioanalytics and Bioprocesses, Am Mühlenberg 13, 14476 Potsdam-Golm, Germany

**Keywords:** polyelectrolyte multilayers, thin films, diffusion, film growth, degradation

## Abstract

Rapid development of versatile layer-by-layer technology has resulted in important breakthroughs in the understanding of the nature of molecular interactions in multilayer assemblies made of polyelectrolytes. Nowadays, polyelectrolyte multilayers (PEM) are considered to be non-equilibrium and highly dynamic structures. High interest in biomedical applications of PEMs has attracted attention to PEMs made of biopolymers. Recent studies suggest that biopolymer dynamics determines the fate and the properties of such PEMs; however, deciphering, predicting and controlling the dynamics of polymers remains a challenge. This review brings together the up-to-date knowledge of the role of molecular dynamics in multilayers assembled from biopolymers. We discuss how molecular dynamics determines the properties of these PEMs from the nano to the macro scale, focusing on its role in PEM formation and non-enzymatic degradation. We summarize the factors allowing the control of molecular dynamics within PEMs, and therefore to tailor polymer multilayers on demand.

## 1. Biopolymer-Based Multilayers

### 1.1. MAIN Principles of LbL Assembly

**Figure 1:**
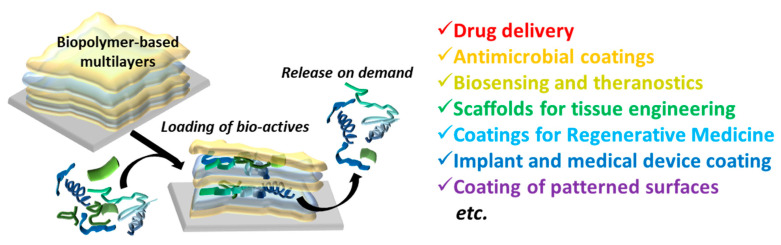
Multilayer assembly made of biogenic polyelectrolytes and their biomedical applications.

The layer-by-layer (LbL) assembly of oppositely charged polyelectrolytes originated in the early 1990s, and since that time, it has gained considerable interest due to its versatility and ability to modulate nanometer control over the film properties (reviews [1,2]). The broad spectrum of usable materials and a choice of coating both flat and particulate substrates provoked exponential growth of research in this field, which has demonstrated the potential of LbL technology for various applications. Particularly, LbL technology presented several advantages for biomedicine: (i) deposition of homogeneous films with controlled thickness, (ii) high loading capacities and controlled release of biomolecules/drugs of various nature, and (iii) coating stability under physiological conditions. This made the LbL method one of the most rapidly growing strategies for generating thin film coatings of biomedical scaffolds [3,4,5,6], patterned surfaces [7,8], medical devices [9,10], implants [11,12], and a range of alternate bioapplications (Figure 1); while multilayer capsules became promising nano- and micro-carriers for drug delivery applications [1,13,14,15,16,17,18,19].

**Figure 2:**
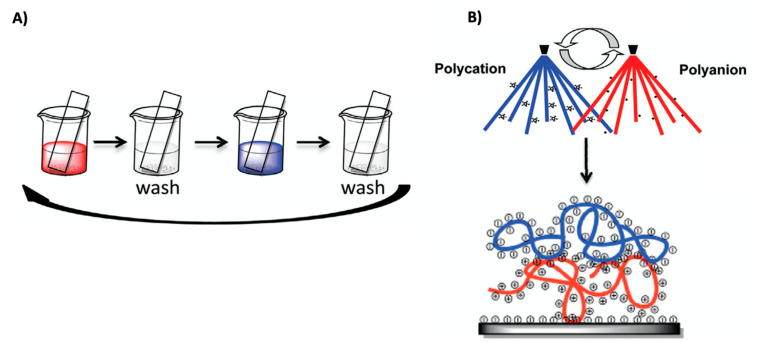
Layer-by-layer approach for PEM fabrication: a negatively charged substrate is alternately immersed (**A**) (or sprayed (**B**), for instance) in polycation and polyanion solutions, respectively. Washing steps are to remove unbound polyelectrolytes. Colour-coding: polyanion (blue); polycation (red). Figure adapted with permission from reference [23], copyright © 2020 Elsevier Ltd.

Generally, LbL fabrication is based on the alternating exposure of a substrate (which can be of nearly any geometry) to positively and negatively charged polyelectrolytes (Figure 2). The three classical methods for applying LbL coatings consist of dip coating, spraying, and spin coating. Each method has distinct advantages and disadvantages that are discussed elsewhere. In brief, alternating dipping of the substrate into the solutions of polyelectrolytes is the most widely used method for LbL deposition. This method is the most simple, robust and versatile among three methods proposed; however, it is also the most time consuming and leaves abundant residual polyelectrolyte from each deposition step, which is an obstacle to its commercialization. Spin coating is based on the rapid evaporation of the solvent from the coating material. Notably, polymer dynamics in spin-assisted PEMs is inhibited due to the stronger binding between spin-deposited polyelectrolytes [20]. Such films are generally thicker than those resulting from the dipping technique; however, spin coating of peculiar 2D substrates and 3D substrates is technically challengeable. In its turn, spray coating is devoid of these shortcomings, although some reports indicate that sprayed samples are more labile to variable external microenvironments [21]. Recently, microfluidics has also been adapted for LbL assembly within microchannels [22].

### 1.2. Biopolymers Used for LbL Assembly

Depending on the nature of the polyelectrolytes, PEMs can be assembled from synthetic, from naturally occurring polymers, or from their mixture. The most important examples of synthetic polyelectrolytes that are used for LbL assemblies and generate cations are poly (allylamine hydrochloride) (PAH) and poly (diallyldimethylammonium chloride) (PDADMAC); typical synthetic polyanions are poly (acrylic acid) (PAA) and polystyrene sulfonate (PSS).

Natural polyelectrolytes, such as components of the extracellular matrix (hyaluronate, collagen, elastin, fibronectin, laminin), proteins (protamine, gelatin), nucleic acids (DNA and RNA) and polysaccharides (which are the most abundant family of natural polymers), have also gained considerable attention as the building blocks for the multilayers [24]. PEMs fabricated from such biogenic polyelectrolytes hold specific bioactivities (e.g., ant-inflammatory [25,26] and osteogenic activities [27]) that are useful for a variety of bioapplications. In some studies, self-assembled structures such as liposomes and micelles were also used as the building blocks for LbL deposition [28,29]. Recent trends in LbL technology include the assembly of hybrid structures composed from polymers, lipids, and nanoparticles [30,31,32,33,34].

Biopolyelectrolytes are intrinsically labile and more difficult to handle than synthetic polyelectrolytes; it makes the design of LbL structures made of biopolymers more challengeable [1]. However, obvious benefits of the use of biopolymers (such as their intrinsic unique biocompatibility, biodegradation, no to low toxicity, high loading capacities and mimicking of the natural cellular microenvironment) [35,36] boost the use of biopolymer-based PEMs for biomedical applications and in other fields such as bioelectronics [37,38], bio-energy [39,40], food packaging/storage [41], etc.

Chemical structures of some biopolymers that have been used for PEM fabrication are sketched in Table 1.

Besides natural polymers, synthetic biodegradable polymers (such as poly aminoacids, poly α-hydroxy acids, etc.) have been widely employed for the fabrication of LbL coatings. The units of these polymers are small natural molecules. In this context, the most classical example is a polymerized form of lysine, polylysine. Polymerization of lysine may occur either via α-carbon or via ε-carbon. Notably, ε-polylysine is a natural polymer produced by the strain of bacteria in the genus *Streptomyces*. α-Polylysine is not found in nature. It is produced synthetically by a basic polycondensation reaction. Depending on the chirality of the lysine’s central carbon, it can be composed of either L-lysines or D-lysines (Table 1) denoted in literature as poly L-lysine (PLL) and poly D-lysine (PDL), respectively; both forms of polylysine are used in PEM fabrication: PLL has long dominated the field as the gold standard and PDL displays similar efficacy and is more resistant to proteolytic degradation [94]. Another example of a synthetic biodegradable polymer that is often used for LbL fabrication of coatings is poly arginine (Table 1).

**Figure 3:**
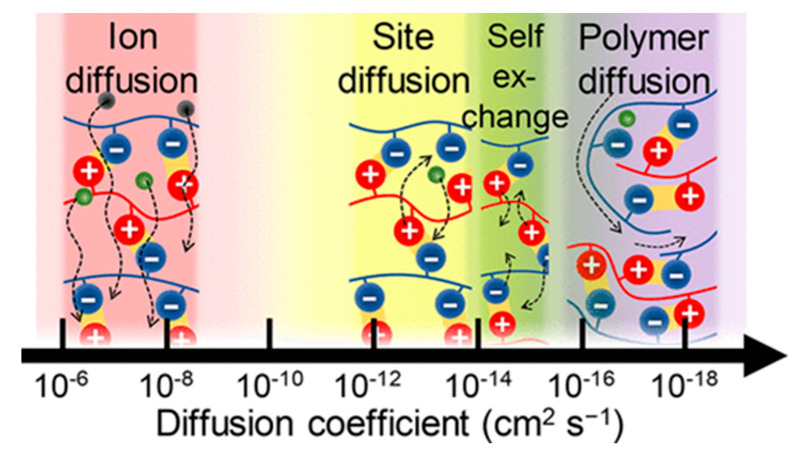
Dynamics within PEMs. Figure taken from reference [97]. Copyright © 2020 American Chemical Society.

Regardless of the nature of the polyelectrolytes, the most predominant interaction in LbL multilayer formation is the electrostatic interaction between oppositely charged polyelectrolytes. In a review [95], it was reported that there is a charge threshold, of which the previously deposited layer on the surface of the template must hold a certain charge density and charge distribution for adsorption to occur. However, additional interactions also contribute to multilayer formation. Generally, the anionic polyelectrolytes employed are composed of a hydrophobic hydrocarbon backbone, with the side groups containing charged groups; therefore, the polymers hold some amphiphilic character, so hydrophobic interactions [96] must also be taken into account for film formation. It has been reported that, from the Gibbs free energy of multilayer formation, for increased numbers of polyelectrolyte pairs, hydrophobic interactions are essential for formation of the multilayers. Furthermore, depending upon the polymers used, hydrogen bonding may also be a decisive factor, especially for use of neutral polymers. All these interactions determine the dynamics of biopolymers in the PEMs. It is commonly accepted to distinguish between the dynamics of polymer molecules that may diffuse inside or in/out the PEMs and the dynamics of polymer chains or binding sites (Figure 3). It is of note that ion diffusion will not be considered in this review. Up to now, analysis and quantification of these molecular dynamics at the nanoscale remain non-trivial and challengeable task. The next section will summarize the progress achieved in this field of research in the last decades.

## 2. Analysis of Polymer Dynamics inside PEMs: Methods

Methodologically, studying the localization and diffusivity of molecules inside the films requires non-invasive techniques that provide high spatial and temporal resolution. So far, confocal-based optical imaging was a pivotal technique used for tracking molecules and probing 3D molecular locations across the PEM matrix [98]. However, conventional confocal laser scanning microscopy of PEMs is not accurate enough in terms of its temporal resolution; this is due to long scanning times and possible loss of the signal due to dye bleaching. To get higher time-resolved information on molecular diffusion, standard confocal imaging has been modified. In this context, the fluorescence recovery after photobleaching (FRAP) approach became the most versatile optical technique for studying molecular transport and dynamics across PEMs [98].

The FRAP approach relies on the bleaching of a selected region of interest within the multilayer film by using a high intensity laser and further monitoring of fluorescence intensity recovery in time in order to track the molecules diffusing from the area surrounding bleached region. The rate of fluorescence recovery gives the information about the diffusion coefficients (D) of fluorescent molecules [99]. In recent years, several studies have used FRAP-based approaches for studying molecular diffusion in PEMs made of synthetic [100,101,102] and bio-polymers [103,104,105]. It has been shown that FRAP cannot be reliably used to study diffusion within ultrathin polymer layers that have thicknesses of a few hundred nm because fluorescence recovery in bleached regions predominantly occurs via diffusion through the surrounding solution layer, but not the PEMs themselves [100]. Luckily, biopolymer-based PEMs are generally thick enough and the diffusion in them can be investigated by classical FRAP approaches. Probing the movement of polymer molecules within condensed polyelectrolyte matrices showed that polymers were significantly (a few orders of magnitude) retarded if compared to their diffusion in the solution. It was also found that polymer molecules in the film occur in two or more distinct fractions that have different diffusivities and are in equilibrium with each other [104].

**Figure 4:**
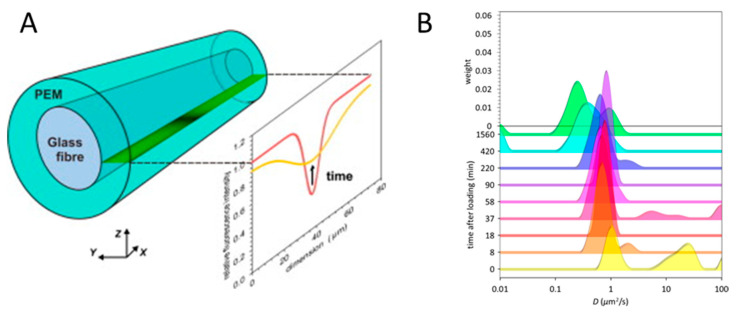
(**A**) Scheme of the microfibre coated with the PEM and the focal plane and FRAP experiment. Figure taken from reference [108], Copyright © 2020 Elsevier Ltd. (**B**) Distribution of diffusion coefficients of CytC loaded into (HA/PLL)_24_ multilayers, where 24 is the number of polymer bilayers. Figure taken from reference [109], Copyright © 2020 American Chemical Society (https://pubs.acs.org/doi/10.1021/acs.jpcb.7b11051).

Notably, these studies are mostly limited to the investigation of lateral transport within the multilayers (e.g., [43,82,105,106]), because CLSM has insufficient optical resolution in a vertical (*z*) plane. Indeed, in practice, the maximum *z*-plane resolution that can be achieved using a conventional confocal microscope is about 0.8 µm. It is lower or of the same order as the typical thickness of biopolymer-based PEMs, that ranges from tens of nm to a few µm. To overcome this limitation and evaluate the diffusion of molecules in 3D, Uhlig et al. developed an elegant approach for a highly resolved side view of PEMs deposited on a cylindrical glass fibre [107]. This approach is based on the preparation of thicker films and its 90° tilting that allows monitoring of vertical molecular diffusion across PEMs using higher-resolution and faster scanning in (*xy*) plane of CLSM (Figure 4A).

In further studies, this approach was mainly applied for studying the diffusion of proteins in HA/PLL PEMs [108,109,110]. It was found that, similarly to the polymers that comprise PEMs, proteins loaded into PEMs also represent several fractions that have different degree of the binding to polymer network and therefore have different diffusivity (Figure 4B) [109].

Forster Resonance Energy Transfer (FRET) is an alternative option used for the investigation of out-of-plane (vertical) molecular diffusion in PEMs [98]. This method is based on the energy transfer between two types of fluorescent molecules labelled by the dyes called a donor and an acceptor dye. Fluorescence intensity in such a system depends on the donor–acceptor energy transfer, of which is extremely sensitive to the distance between the fluorophores and therefore can be used to evaluate the proximity of labelled molecules as they diffuse (review [111] and references therein). FRET approaches allow the probing of polymer dynamics in the vertical direction within the 1–10 nm range that is significantly higher than the spatial resolution of FRAP. However, this method is associated with multiple limitations inherent to the physical processes underlying FRET itself [111]. The main drawback is the low signal-to-noise ratio of FRET signals originating from the loss of energy associated with the FRET process. The use of two or more fluorescent labels that are sensitive to local changes in pH, ionic strength, temperature, oxidation, refractive index, etc., complicates the interpretation of FRET data [111,112]. As a result, there are only a few reports on evaluation of molecular dynamics in PEMs using FRET. Thus, it has been used for the evaluation of the vertical transport and its restriction in synthetic PAA/PAH PEMs compartmentalized with different barrier layer PEMs [113]. Considering biopolymer-based films, vertical diffusion of CHI fluorescently labelled with either donor or acceptor dyes within CHI/heparin PEMs was investigated by means of FRET, revealing that these PEMs have a highly interpenetrated structure with blurry boundaries between each layer. The high dynamics of CHI/heparin PEMs was correlated with exponential-like growth of the film [69]. Similarly, the dynamic nature of DNA-based multilayer films has been demonstrated using FRET [114].

Fluorescence Correlation Spectroscopy (FCS) is another optical technique that provides valuable information on the polymer diffusion and exchange. This method provides information about molecular transport across the PEMs at temporal scales ranging from sub-microseconds to several hundreds of milliseconds. As well as other confocal-based techniques, FCS requires fluoresce labelling of tracked molecules [98].

Complementary to FRAP, that (in its classical set-up), allows us to probe chain diffusion in the direction parallel to the substrate, neutron reflectometry may provide some additional information on the interlayer (perpendicular to the substrate) diffusion of the polymers. Thus, combination of FRAP and neutron reflectometry applied to poly (2-(dimethylamino)ethyl methacrylate)/poly(methacrylic acid) PEMs revealed that polymer chain mobility in such films is highly anisotropic [115]. In addition, advanced electrokinetic characterisation of PEMs provides useful information on electrohydrodynamics of diffuse soft planar interfaces in multilayer films, of which can be used for indirect evaluation of molecular dynamics within PEMs [116]. Further to this, atomic force microscopy (AFM) paired with Raman and IR spectroscopies have recently emerged as techniques for characterising polymer-based materials at the nanoscale [117].

Along with experimental methods, some recent studies apply theoretical approaches for studying molecular dynamics in PEMs. Thus, bead-spring models make it possible to simulate intermixing of polymers within the PEMs depending on charge density and the degree of polymerization. First, molecular dynamics simulations of PEMs were performed using coarse-grain or implicit-solvent models, and therefore they did not allow the assessment of the effects of polymer dynamics on macroscale properties of PEMs [118,119]. However, some recent simulations have been applied for the HA/PLL system at nearly atomistic resolution, making it possible to quantitate the effects of temperature, salt, and the protonation state of the polymers on their stability and PEM dynamics [120,121]. Later, molecular dynamics simulations of the system consisting of PARG and casein helped to decipher the role of hydrogen bonds in PARG–casein interactions, which, along with their impact on the growth mechanism of the film, were correlated with FTIR and polymer conformation analyses [93]. Molecular dynamics simulations in combination with single-fluorophore visualisation techniques may prove a powerful tool for the study of molecular dynamics in PEMs [122].

Altogether, these methods allow the estimation and, in some cases, the quantification, of the diffusivity and dynamics of polymers in PEMs; ongoing research in this field will enrich and improve our understanding of polymer dynamics in future. However, nowadays it is already obvious that polymer dynamics plays a central role in PEM fabrication, determines the properties of PEM and guides their non-enzymatic degradation, if PEMs are exposed to certain microenvironment. Generally, this is valid for all PEMs regardless of the nature of polymers used for their assembly. However, intrinsic complexity, sensitivity to the microenvironment, and (often) polydispersity of biopolymers favour the role of polymer dynamics in biopolymer-based multilayers. The next sections will consistently bring together the knowledge of the role of polymer dynamics in PEM growth, PEM mechanical properties and PEM degradation. Notably, loading/release performance of PEMs is beyond the scope of this review, which is focused on plain PEMs.

## 3. Biopolymer Dynamics at the Macroscale

### 3.1. Polymer Dynamics and PEM Growth

The growth of PEMs is something that has been widely discussed in the literature. Typically, two different growth regimes are observed when assembling PEMs; these are the linear and non-linear (“exponential”) growth regimes. The former involves a film that grows linearly with each additional polymer deposition step i.e., the thickness increment by which the PEM increases by remains constant throughout the multilayer build-up. The latter involves a film thickness that increases exponentially with each polymer deposition step. Many synthetic polymers form linearly growing PEMs, one extensively studied pair is the PSS/PDADMAC system [123,124]. Linear growth can only take place if the charge of the newly added polyelectrolyte overcompensates that of the surface it is adsorbed upon [125]; this may occur if there is some additional affinity the freshly added polymer has to the pre-adsorbed polyelectrolyte at the surface, such as hydrophobic interactions or van der Waals forces.

**Figure 5:**
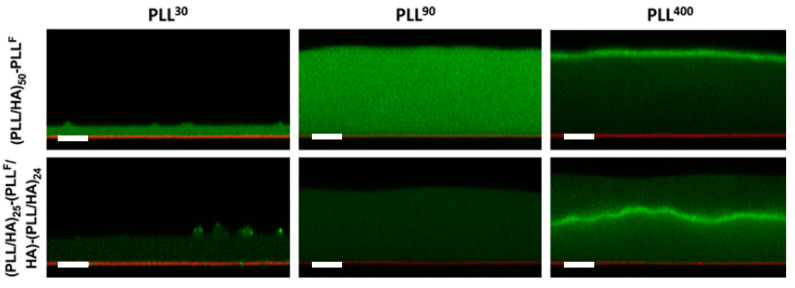
Examining PLL mobility within (PLL/HA)_50_ films using CLSM and FRAP experiments: cross-sectional CLSM images of (PLL/HA)_50_-PLL^F^ (top) and (PLL/HA)_25_-(PLL^F^/HA)-(PLL/HA)_24_ films (bottom) assembled with PLL^30^, PLL^90^, and PLL^400^ (left to right). Green indicates PLL^F^ and red indicates the glass substrate. The scale bar is 10 μm. Figure taken from reference [129], Copyright © 2020 John Wiley and Sons Ltd.

The exponential growth of PEMs is much more dependent upon the polymer dynamics within the multilayer; it has been shown that even PSS/PDADMAC PEMs exhibit exponential growth at higher ionic strengths, where the PEM behaves similar to a fluid rather than a glassy solid [125]. It is widely thought that the mechanism in which exponential growth occurs involves the whole of the PEM. Exponential growth can only occur if at least one of the polymers constituting the PEM is able to diffuse in and out of the film [126,127]. For example, if the polycation is the diffusive species within the PEM, it will be able to diffuse out of the film when the film is in contact with polyanion-containing solution, and form complexes with the polyanion chains at the surface (forming a new layer). Therefore, the thickness increment added can be said to be proportional to the number of polycation chains able to diffuse out of the PEM when in contact with polyanion-containing solution, which is also proportional to the total PEM thickness. This leads to the exponential growth regime. This was first shown by Picart et al. (2002) [128], demonstrating that within HA/PLL PEMs, PLL was able to diffuse throughout the whole PEM to interact with HA at the surface to form polyelectrolyte complexes, contributing to a new layer, and leading to an exponential increase in PEM thickness. Later, it was demonstrated that the distribution of PLL within PEMs depends on its dynamics, which is correlated with the degree of its polymerization (Figure 5) [129]. 

**Figure 6:**
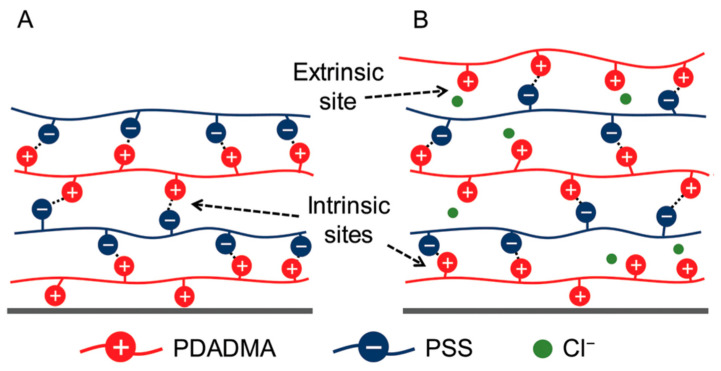
(**A**) PSS/PDADMAC multilayers containing only polyion-polyion interactions (i.e., intrinsic charge compensation) and (**B**) the same multilayers but overcharged or overcompensated; also containing extrinsic polyion-counter ion interactions. Figure taken from reference [130], copyright © 2020 Royal Society of Chemistry.

The charge densities of biopolyelectrolytes play a profoundly important role in stable PEM formation. Two different types of PEM charge compensation are typically observed: extrinsic and intrinsic charge compensation. Extrinsic charge compensation refers to any excess polyelectrolyte charge being compensated via counter ions arising from salt (i.e., Na^+^ and Cl^−^), where intrinsic charge compensation refers to the balance of charge between pre-existing polyelectrolytes within the PEM [130] (a scheme of which is presented in Figure 6). Due to their inherently low charge densities, one can expect that the majority of biopolymer-based PEMs will exhibit exponential growth, making extrinsic charge compensation much more important within these systems. Many studies have depicted the growth regimes of biopolymer 2D PEMs; multiple studies are present on, arguably, the most studied biopolymer PEM, the exponentially growing HA/PLL system, which typically forms a viscous, hydrogel-like film [131]. Others include PEMs such as the highly studied CHI and PLL systems, both of which have been previously paired with HA [22,48], ALG [61,132], HS [68,83], CS [72,81], and PGA [133]. Each of these systems have been shown to grow exponentially. Lundin et al. (2009) [69] demonstrated that CHI is able to diffuse throughout the whole film (7 bilayer film), through FRET and total internal reflection fluorescence (TIRF) approaches, when in combination with HS. Furthermore, Mutschler et al. (2017) [88] conducted a systematic study comparing the exponential character of PARG paired with ALG, CS, HA, HS, PGA and PSS within PEMs. It was observed that all combinations resulted in the exponential growth regime, apart from PSS. Therefore, it can be said the exponential character of the film increases with decreasing polyion–polyion interaction. The HA/PARG reaction enthalpy was endothermic, and hence held the weakest interaction; this suggests a strongly exponential-like growth regime, as demonstrated by Laugel et al. (2006) [134]. It can be said that the weaker the interaction between the two polyelectrolytes, the easier the diffusion is throughout the film, which is the cause of exponential growth. A strong exothermic interaction between two polyelectrolytes will result in a much more favourable interaction for the polymers, and hence a tightly knit polymer network within the PEM, leading to the linear growth observed for PSS/PARG, PSS/PDADMAC [124], PSS/PAH [135], and other synthetic systems. However, biopolymer pairs which possess linear growth regimes are possible; Radeva et al. (2006) [78] reported linearly growing CHI/CMC PEMs, when formed using highly charged CHI and weakly charged CMC at low ionic strengths, the effect of which will be discussed later. Aside from these studies, it can be relatively difficult to obtain a stable biopolymer PEM; their lower inherent charge densities may result in too weak an interaction for biopolymer-based PEMs to form at all, especially at physiological conditions. For example, HA has been previously paired with COL [46], but the PEM proved to be unstable at physiological pH due to protein reorganisation and changes to the degree of ionisation of HA at these conditions. However, the film became stable upon cross-linking the polymers within the film.

**Figure 7:**
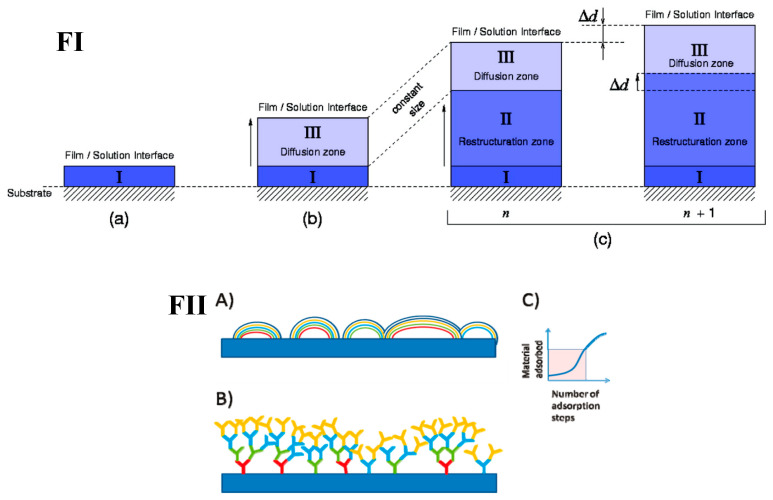
Models of film build up. **(FI**) Model of the restructuration zone; (**a**) initial film build-up upon the substrate, (**b**) the development of the diffusion zone within the film, and (**c**) the formation of the re-structuration zone, including the case where the number of deposition steps increases from *n* to *n +* 1 (**FII**) The substrate for film fabrication is shown as a horizontal slab. The film resulting from successive polymer adsorption steps is shown via different colours, each representing a polymer layer. (**A**) Island model, (**B**) Dendritic model, and (**C**) Approximate growth of material deposited. (**FI**) is taken from reference [139], Copyright © 2020 American Chemical Society. (**FII**) is taken from reference [137], copyright © 2020 American Chemical Society.

However, there comes a point during PEM build-up in which the exponential growth switches to linear. How this transition occurs is still debated within the literature. An “island” model has been proposed, where, firstly, islands grow upon a substrate (their growth is exponential); eventually they will coalesce and form a uniform film across the substrate [136]. A model based on the dendritic nature of linear polymers was also suggested, at which the transition point occurs due to steric hindrance of the polymer chains during the exponential build-up, finally resulting in a constant number of binding sites [137] (Figure 7FII). A model proposed by Hübsch et al. (2004) [135] stated that once the PEM reaches a critical thickness at which neither of the polymers are able to diffuse through the whole PEM when in contact with the alternate polymer solution, the diffusive species that will contribute further towards the growth of the PEM will be the zone just below the PEM–solution interface. Therefore, the number of polymer chains able to diffuse in and out of the PEM becomes constant as this zone will remain a constant thickness upon further addition of new polymer layers, leading to the linear growth regime. Salomäki et al. (2005) [138] proposed a similar model, proposing a diffusion-limited mechanism also, where the PEM reaches a thickness at which the rate of diffusion is too low for exponential growth to continue. Moreover, it is put forward that as the PEM thickness increases, a “restructuration zone” develops due to the rearrangement of polymer chains as the build-up continues (Zone II in Figure 7FI). This zone holds a much higher polymer density compared to the rest of the film, in which polymer diffusion is greatly limited. This was first developed by Porcel et al. [139,140], in which, as the exponential build-up continues, eventually this restructuration zone will develop, within which polymer diffusion is so hindered that the part of the film contributing to PEM growth reaches a constant thickness (Zone III in Figure 7FI).

From this point on, the film grows linearly with deposition steps. HA/PLL films have been shown to exhibit an exponential-to-linear transition point between twelve and eighteen bilayers [139], and CHI/PGA have been reported to transition around nine deposition cycles [133]. However, this number may vary with build-up conditions able to alter the diffusion of polyelectrolytes through the PEM (Such as temperature [141], ionic strength and pH).

Certain microenvironmental changes can drastically influence the growth and build-up regimes of PEMs. This is one of the reasons why biopolymer-based multilayer films have become attractive drug delivery/reservoir systems, due to their ease of tunability and respective properties. As mentioned above, biopolymers are much more dynamic and possess low charge densities, making biopolymer-based PEMs highly sensitive to their microenvironment. This gives us the opportunity to alter the microenvironment during PEM build-up in order to tune their growth dynamics. In this review, we consider three main factors—the temperature, the pH and the ionic strength—that influence the growth of PEMs.

#### 3.1.1. Effect of Temperature

**Figure 8:**
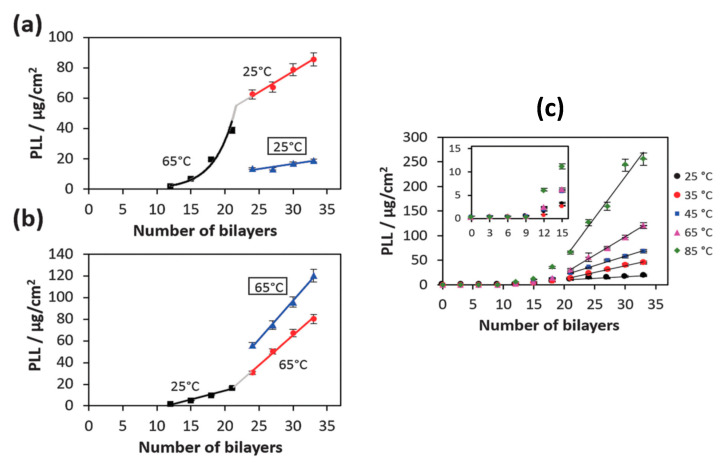
(**a**) Temperature change from 65 °C to 25 °C; (**b**) Temperature change from 25 °C to 65 °C at the 21st bilayer. The blue triangles represent film preparation at 25 °C and 65 °C without the temperature change after 21 bilayers. (**c**) Mass coverage of PLL in the HA/PLL film formed at different temperatures. The inset shows the enlarged growth profile until 15 bilayers. Figure taken from reference [141], copyright © 2020 Published by the PCCP Owner Societies.

In the study [141], the effect of temperature on the transition point from exponential to linear growth of HA/PLL PEMs was investigated. It was found that the transition point could be shifted from 12 to 21 bilayers. The increase in preparation temperature (to a maximum of 85 °C) increased the polymer mobility, and therefore the diffusion rate of PLL, allowing PLL to diffuse through the entire PEM even when the total PEM thickness increased. It is also inferred that these multilayers grown at higher temperatures are more porous, allowing for less-hindered diffusion. This was demonstrated through temperature changes during PEM build-up at 21 bilayers, either from 25 °C to 65 °C, or vice versa (Figure 8). When increasing the temperature during PEM build-up, the film grows slower compared to when it is fully grown at a higher temperature to begin with. One can expect a difference in PEM structure as the temperature changes through initial build-up stages as the polymers will have higher thermally induced motion, and as such the polyion–polyion interactions will be weakened and the film is more porous. For instance, it has been shown that even in synthetic PEMs that an increase in temperature can alter the growth regime of the film, extending the exponential growth region at the beginning of film build-up, when using the polymer pairs PSS/PDADMAC and PSS/PAH, with an increase in deposited bilayer mass [138].

#### 3.1.2. Effect of pH 

**Figure 9:**
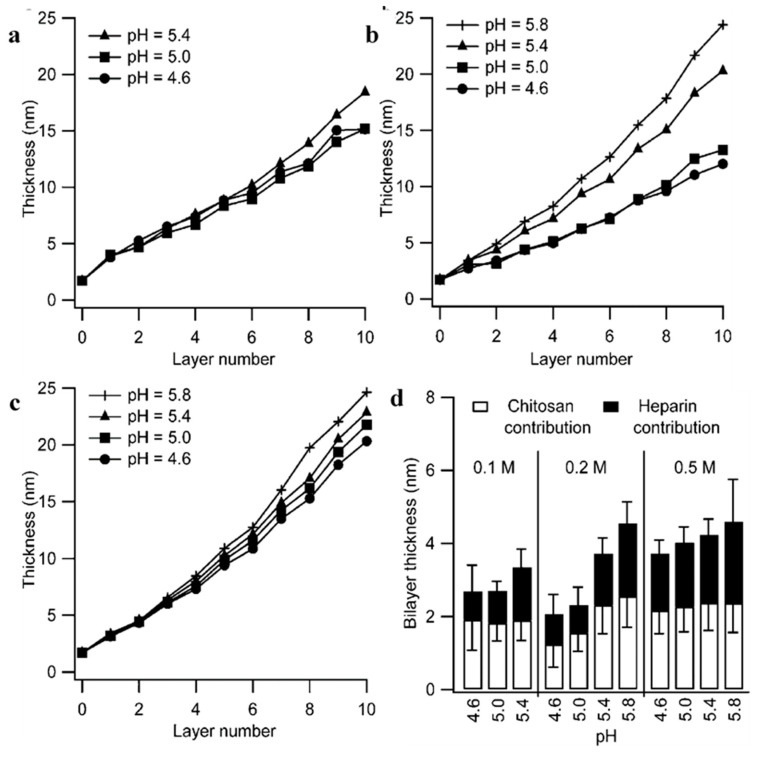
PEM thickness obtained from in situ Fourier transform-surface plasmon resonance (FT-SPR) data as a function of layer number and pH for 0.1 M buffer (**a**), 0.2 M buffer (**b**), and 0.5 M buffer (**c**). Odd numbered layers are CHI and even-numbered layers are HS. (**d**) Average incremental bilayer thickness of PEM at different buffer conditions as a function of pH. Figures taken from reference [71], Copyright © 2020 American Chemical Society.

Controlling the pH during PEM build-up is also of crucial importance, especially for weak polyelectrolytes in which their charge densities can be easily tuned. Yuan et al. (2007) [58] reported the effect of build-up pH upon CHI/ALG multilayer films; the pH of the ALG solution was altered while CHI remained constant at pH 3. The thickness of ALG layers decreases with increasing pH, this is due to the increased ionisation of the ALG chain, causing the chain to become more extended, to give thinner layers. Changing the polyion solution pH also gives the opportunity to tune the surface composition; In the same study, Yuan et al. demonstrated that increasing the ALG solution pH changes the conformation of the CHI layer on which it is deposited, inducing more globular complexation between the two polyions, as well as aggregation of the CHI molecules due to less intra-chain repulsion at higher pHs. This change in conformation also resulted in the increase in relative thickness of the neighbouring CHI layer. These results both support and build upon the results of Bieker and Schönhoff (2010) [142], in which the thickness of the PEM of two weak polyelectrolytes was significantly altered within a narrow pH range. It was found that, at either low or high pH, where one of the polyions is more ionised than the other, interpenetration between polymer layers occurs and the growth becomes exponential. This is opposed to a relatively equal ionisation degree between the two polymers at an intermediate pH, which gives very defined layers, and hence, the linear growth regime. However, at pHs where there is a dramatic difference in ionisation degrees, this interpenetration stops and asymmetric growth occurs, causing the PEMs to grow linearly again. Indeed, the resulting growth behaviours are governed by the degree of ionisation of polyelectrolytes at certain pHs [143]; depositing each layer at pHs where the polyelectrolytes are partially ionised and in coiled conformations (close to their pKa), i.e., HA (pH 2.9) and PLL (pH 9.5), will result in thicker layers being deposited [144]. Again, Burke et al. (2003) [145] reported the build-up of HA/PLL films at different pHs; pH 7 resulted in the thinnest PEMs where pH 9 gave the thickest. This was due to the partially ionised and coiled conformation of PLL, in comparison to HA at pH 9, resulting in higher adsorbed quantities of PLL; where, at pH 7, both polyelectrolytes are almost fully ionised, resulting in flatter conformations. This observation was also noted by Barrantes et al. (2012) [84], in which it is proposed PLL and HS adopt a “flat” conformation at more acidic conditions (pH 5.5), and PLL is thought to adopt an α-helical structure at higher pHs (7–8.5, depending on the substrate used for deposition) due to its reduced charge density. This results in the exponential growth of HS/PLL PEMs as the lower charge density of PLL allows for easier diffusion through the entire film. Moreover, Rocha Neto et al. (2019) [48] observed the opposite effect, reporting thinner deposited layers of both HA and CHI at pH 5 due to the high charge densities, and hence, rigid conformations of both polyelectrolytes. This results in lower surface roughness of the film due to a lower concentration of randomly coiled HA and CHI chains penetrating the surface. The effect of pH upon the incremental bilayer thickness at different ionic strengths has also been studied within HS/CHI PEMs. As the pH of CHI approaches its pKa (6.46–7.32 for NH_2_ groups) from a more acidic pH, it becomes increasingly less ionised; as such, it adopts are more coiled conformation upon the pre-adsorbed HS surface, as well as adsorbs in higher quantities. Therefore, with increasing pH from 4.6 to 5.8, there is an increase in bilayer thickness at all ionic strengths studied [71], as seen in Figure 9. The authors attributed the increasing HS layer adsorption with increased pH to the increasing mass of CHI adsorbed, so a larger amount of HS is needed for charge reversal.

#### 3.1.3. Effect of Ionic Strength 

The ionic strength of the solution can profoundly affect the assembly of PEMs, especially those in which extrinsic charge compensation is of great importance (i.e., biopolymers). Boddohi et al. (2008) [71] reported an increase in HS/CHI film thickness with increasing ionic strength within the polyelectrolyte dipping solutions, this was attributed to the effective electrostatic screening of charges along the HS and CHI chain. This will give rise to less intra-chain repulsion and result in more coiled conformations of polymers resulting in thicker deposited layers, as seen in Figure 9. Moreover, Lundin et al. (2011) [70] reported similar results, and observed the exponential growth regime of PEMs regardless of the ionic strength used. Interestingly, Richert et al. (2004) [146] demonstrated the transition from exponential to linear growth when decreasing the ionic strength from 0.15 M to 10^−4^ M, and linear growth for a (HA/CHI)_8_ film (built at 10^−4^ M) built atop an exponentially growing (HA/CHI)_9_ film (built at 0.15 M). At concentrations of 10^−4^ M, only PEM islands of HA/CHI films could grow upon the substrate but appeared to grow linearly. At higher ionic strengths (0.15 M), the islands were only present for the first deposition steps, until they coalesce to form a uniform film—the higher the salt concentration, the earlier this coalescence. The increased ionic strength screens the charges along the polyelectrolyte chain, reducing HA-CHI interaction and allowing for easier diffusion of CHI through the entire film compared to lower ionic strengths (where only islands may form), resulting in the final thicker PEM.

### 3.2. Polymer Dynamics and Physicochemical Properties of PEMs

When discussing biopolymer dynamics within PEMs at the macroscale, these include phenomena such as: shrinkage, swelling and de-wetting of the PEM film. Shrinkage of PEM structures involves the reduction in total size of the film due to the partial increase in polymer dynamics within the multilayers. Shrinkage of multilayers is especially prevalent in PEM capsules; some sort of environmental change is generally required to induce capsule shrinkage (i.e., pH [147] or temperature [90,148]), either some sort of segmental change within the multilayers via a change in their state from a glassy solid to fluid-like [149], charge density or kinetic energy for instance, and these will be dependent upon the inherent physical properties of the polymers in question (i.e., their glass transition temperatures or pKa). Both swelling and shrinkage of PEMs can influence multiple film properties, such as their thickness, permeability and rate of release, and this leads to great implications in the field of drug delivery [150].

The swelling of PEM structures typically occurs when the film is in contact with a penetrating liquid (e.g., water); this liquid diffuses within the polymeric network and causes the system to swell. For instance, the 2-fold swelling of ALG/CHI PEMs when in the wet state as reported by Caridade et al. (2013) [151] upon immersion in PBS buffer solution. In this case, the liquid solvent penetrates the PEM via pre-existing pores/voids within the film. Swelling or shrinkage of PEMs may also be induced via changes in the local microenvironment, such as changes in temperature, pH or ionic strength [152]. Controlled shrinkage and swelling upon these chemical [153,154,155,156,157,158,159] or physical stimuli (e.g., infra-red light [32,160,161,162,163], magnetic field [164]) has been mainly developed for polyelectrolyte capsules and biocoatings applied for drug delivery applications.

#### 3.2.1. Effect of Temperature 

Once the PEMs are formed, external microenvironmental changes can influence the structure and properties of the PEM. Diamanti et al. (2016) [61] demonstrated the temperature-induced annealing of PLL and ALG multilayers after their formation; the increase in temperature results in the re-organisation of the polymers within the PEM to form complexes and achieve maximal charge compensation. This is shown via AFM analysis of the surface topography, the higher the temperature of annealing, the smoother the surface, while also changing the wettability of the surface from hydrophilic to hydrophobic. However, partial erasure of the film is observed at higher temperatures (80 °C). Similar studies have taken place within DS/PARG PEM capsules, the increase in temperature increased the kinetic energy of the polymers, resulting in the annealing of the multilayers, to give a more compact capsule shell [84]. Furthermore, Hellwig et al. (2018) [165] demonstrated the softening of HA/PLL PEM films, attributing this to an increase in temperature from 10 to 50 °C resulting in the kinetic energy increase and induced thermal motion of polymers. This in turn weakens the PEM complexation sites and lowers the indentation modulus. PEMs built-up of GEL and poly(galacturonic acid) (PGL) built at 20 and 25 °C both resulted in the disassembly of the film when increasing the temperature to 32 and 37 °C, respectively. This treatment left 10% and 30% of the hydrated mass upon the substrate after temperature cycles, respectively. This was due to the disassembly of the hydrogen bonding network between the polymers at elevated temperatures, as well as the change of conformation of GEL from a triple-helix structure (where hydrogen bonding is a dominant interaction) to random coils, where hydrogen bonding is less prevalent [64].

#### 3.2.2. Effect of PH 

The stability of the PEM is also highly dependent upon the pH of the surrounding solution as the charge of the polyelectrolyte at a certain pH is dependent upon its pKa. Biopolymers in particular are very sensitive to changes in pH due to their already weak electrostatic properties. Generally, there is a critical pH at which the PEM loses integral stability and the film undergoes full or partial dissolution. This may arise from the drastic change in charge balance within the multilayers upon significant change in the pH, resulting in possible repulsion, altered diffusion of counterions and changes in osmotic pressure within the film.

Thus, pH-dependent swelling will be the result of the differences between biopolyelectrolytes’ pKa. For example, GEL/PGL PEMs undergo a swelling/de-swelling phenomenon upon alteration of the pH (the pKa of poly(galacturonic acid) is ~3.5 and the isoelectric point of GEL is 8.8). As such, at pH 5, GEL will hold a positive net charge and PGL will be fully ionised at pH 7; therefore, changes in pH will result in drastic changes in the degree of ionisation of both polymers [64]. Changing the charge balance within the PEM will lead conformational changes to the polymers in question, and hence to the swelling/shrinkage phenomena observed in a given pH range.

**Figure 10:**
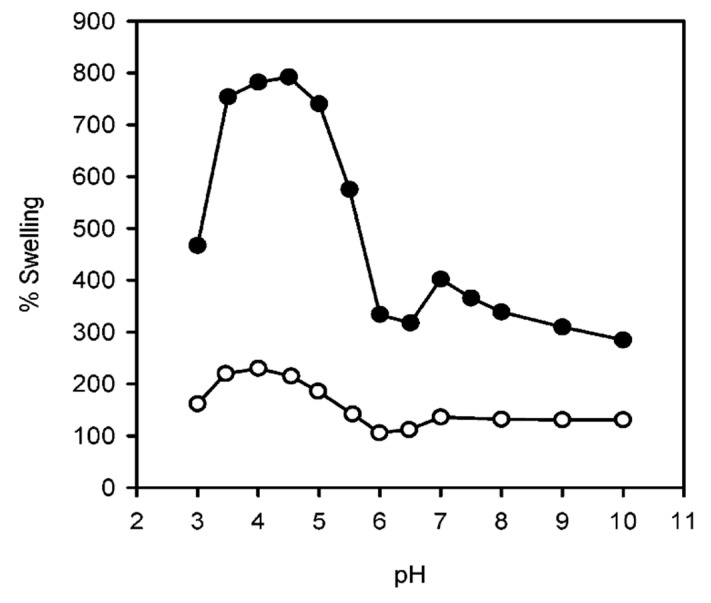
Dependence of the degree of swelling for (PLL/HA)_75_ films assembled at pH 5 (°) and 9 (●). Figure taken from reference [145], Copyright © 2020 American Chemical Society.

For instance, Balabushevich et al. (2003) [166] demonstrated the pH-dependent swelling of DS/protamine capsules templated upon melamine formaldehyde cores. When increasing the pH range from 3–5 to 7–8, the diameter of the capsules grew from 8 μm to 9–10 μm, respectively, leading to an increase in the amount of peroxidase encapsulated. Much greater swelling coefficients of up to 5-fold with increases in ionic strength [167], and 8-fold with pH dependence [145], have been reported for HA/PLL PEMs. The increase in degree of swelling with increasing pH of assembly is explained in terms of the biopolyelectrolyte conformation within the film for PEMs built-up in different pH environments. For instance, for films built at pH 5, HA is near-fully ionised and PLL is completely ionised, the conformation in which they are adsorbed is flatter than at pH 9, where PLL is very weakly charged and will hold random coiled conformations. Polymers within the PEMs can hence hold higher segment mobility upon environmental changes, leading to greater swelling capacities via the diffusion of ions into the environmentally induced voids within the PEM, bringing higher osmotic pressure to induce the swelling phenomenon. This greater degree of swelling for pH 9 assembled PEMs can be seen in Figure 10. This high level of control over swelling capacity may hold important applications in the fields of drug delivery and tissue culture, whether via tuned PEM stiffness or release dynamics through changes in permeability in certain pH environments.

**Figure 11:**
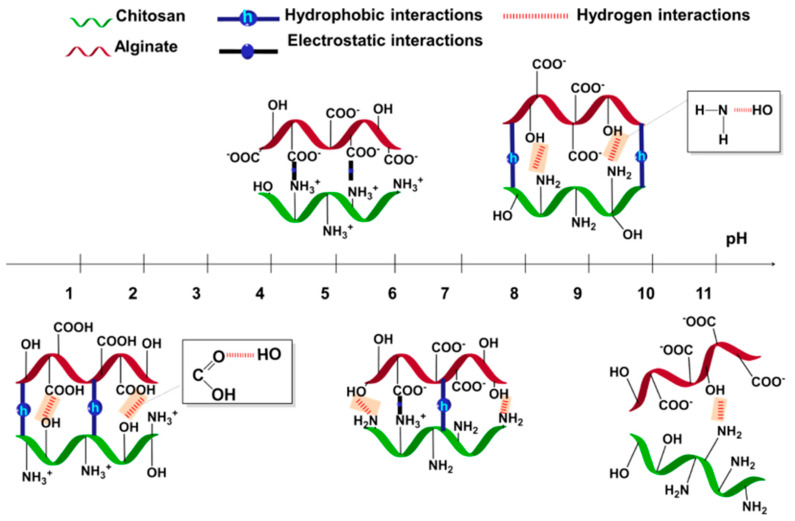
Schematic demonstrating the effect of pH on the molecular mobility of multi-layered films, considering the different intermolecular interactions behind them. Figure taken from reference [168], copyright © 2020 American Chemical Society.

Considering the ALG/CHI PEM [168], the film is stable in the pH range 3–9 and undergoes reversible swelling/de-swelling within this range. Above pH 9, around 10–13, the critical pH is reached, and full or partial dissolution takes place. This is due to the dramatic increase in electrostatic repulsion between ALG chains at pH > 10, resulting in an increase in osmotic pressure (due to the influx of counter ions penetrating the PEM) that is no longer counterbalanced by hydrophobic interactions and hydrogen bonding, a schematic outlining the molecular effect of pH upon these PEMs is seen in Figure 11. Moreover, PEMs consisting of CHI and TA built at pH 5 underwent dissolution when exposed to a buffer of pH 2, this was because of the dramatic reduction of polyion pairs between the two polymers. This was also the case at pH 8.5, where the amine groups of CHI become uncharged and cannot interact as strongly with the phenolate groups of TA—resulting in dissolution of the film [75]. Szyk-Warszyńska et al. (2014) [169] reported similar results in which multilayers formed of PARG and casein were completely removed from the substrate at pH 2 due to the charge reversal of casein from negative to positive at such an acidic pH. However, at pH 11, only the last layer of the film is removed, suggesting the beneath layers are intermixed and stable at these conditions. Mucin/CHI films have been shown to lose adsorbed mass after acetic acid buffer and water (both pH 7) solutions, decreasing the protonation of CHI and reducing the number of polyion pairs within the PEM, resulting in partial dissolution [79]. Further studies with similar findings come from Chen et al. (2016) [51], who found that increasing the pH of HS/COL PEMs from 6.5 to ~ 7 resulted in the gradual degradation of the film, even under static conditions, attributed to the reduction of NH_3_^+^cationic sites on COL.

#### 3.2.3. Effect Ionic Strength 

Increasing the ionic strength of the environment in which a PEM is based may have profound effects on its structure and stability. As discussed earlier, the increase in ionic strength will result in the addition of counter ions which are able to diffuse throughout the pre-built film and reduce the extent of polyion pairing between polyelectrolytes. HA/PLL films built-up at 0.15 M NaCl pH 7.4 have been shown to hold a critical ionic strength of ~0.3 M NaCl [167], that is, the ionic strength at which the biopolyelectrolyte charges are screened via counter ions to the extent to which the PEM is no longer stable and undergoes partial or complete dissolution. Above this critical value, the films undergo full or partial dissolution via a de-wetting mechanism. Svensson et al. (2006) [79] treated Mucin/CHI films with a saline acetic acid solution (150 mM NaCl) and observed a decrease in the total adsorbed mass within the film. This was attributed to the electrostatic screening of polyelectrolytes within the PEM, and reduced polyion pairing. This same effect is observed within HA/PLL PEMs, in which the increased ionic strength resulted in the softening of the PEM [165]. Polysaccharides paired with biodegradable polyurethane (PU) resulted in stable PEMs below 30 mM NaCl; an increase beyond this concentration resulted in the dissolution of PU/CMC and PU/ALG films as a result of the diffusion of counter ions throughout the film, disrupting the polyion-polyion pairing. PU/HA films however, remained stable—the authors attributed this to the presence of amide groups along the backbone of HA, being non-ionic hydrophilic groups to contribute to the salt tolerance of the system [170].

#### 3.2.4. Cross-Linking of PEMs 

Due to the relatively weak interaction between biopolymers within PEMs, it can also be preferable to cross-link films. Cross-linking can have profound effects on the stability of PEMs. Thus, it is known that cellular adhesion correlates with the mechanical properties of the films showing that softer films are weakly adherent to cells [171]. Cross-linking improves cell adhesion and proliferation, typically due to the change in PEM mechanical properties [172]. This technique has been employed in a variety of 2D and 3D biopolymer-based PEMs with a variety of cross-linking agents. The agents typically seen in the literature include genipin (GnP) (a natural cross-linker), glutaraldehyde [173] and 1-ethyl-3-(3-dimethylaminopropyl) carbodiimide (EDC) with N-hydroxysuccinimide (NHS). Additionally, alternate techniques have also been employed to cross-link films without using cytotoxic materials (e.g., glutaraldehyde), such as using calcium ions to induce gel-forming ability of alginic acid in ALG/PARG PEMs [60] in order to dramatically alter the permeability and mechanical properties of the film.

**Figure 12:**
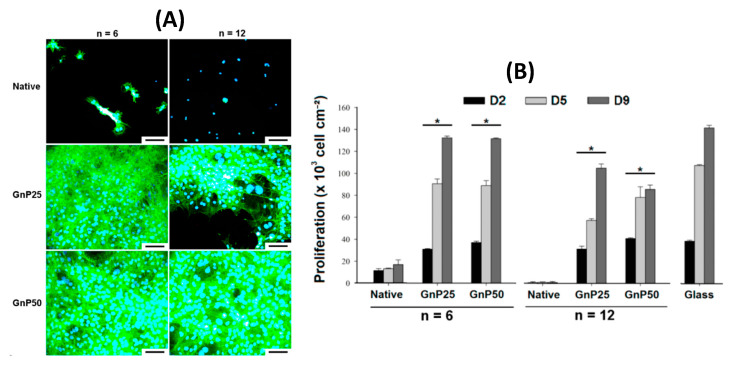
Fluorescence images of MC3T3-E1 cells after 10 days of culture atop native, GnP25- and GnP50-crosslinked PEI/(CS/PLL)n films in standard medium (blue: DAPI-labelled nuclei; green: FITC-labelled actin network) Scale bars are 100 μm (**A**). Proliferation of cells upon the various films, as measured by Alamar Blue assays, after 2, 5, and 9 days (D2, D5, D9, respectively) of culture (**B**). * represents *p* < 0.05 with respect to the corresponding native films. Figure taken from reference [81], Copyright © 2020 American Chemical Society.

In 2014, Gaudière et al. [81] demonstrated GnP (Either 0.25 or 0.50% (w/w), denoted GnP25 and GnP50, respectively) cross-linked (CS/PLL)_n_ (n = 6 or 12) PEMs hold much greater cell adhesion and proliferation properties when compared to native, (non-cross-linked) films, when using MC3T3-E1 preosteoblasts. The results of which were very similar compared to glass substrate controls (which can be observed in Figure 12B), with surface colonisation of cells being better overall compared to native films. Interestingly, with increasing bilayers, the cells displayed extended filopodia, following the dense cross-linked domains on the surface, following the valleys upon the surface of the film, where the film is stiffer. These results are similar to that of cross-linked HA/PLL PEMs (12 layers), in which Schneider et al. (2006) [174] achieved enhanced chondrosarcoma cell adhesive properties with increasing concentration of the cross-linking agent (EDC), resulting in stiffer films. Enhanced cell spreading was also observed upon EDC-cross-linked HA/PLL PEMs, of which the authors believe the change in surface properties (i.e., wettability and surface potential) as well as the increase in Young’s modulus play key roles in guiding cellular behaviour atop such films [175]. 

However, contradictory to Schneider, it was found that GnP cross-linked CHI/HA PEMs [56] do not give rise to an increase in MC3T3 cell adhesion, regardless of the terminating layer, bilayer number or GnP concentration. For native PEMs, increasing bilayer number significantly reduced cell adhesive properties compared to that of 3 bilayers; the authors attributed this to the initial formation of PEM islands upon the glass substrate at low bilayer numbers, allowing cells to adhere to the substrate, also. Indeed, CHI/HA PEMs give rise to thick (~400 nm), highly hydrated viscoelastic films, which are not preferable for cell adhesion and proliferation. Furthermore, it is inferred from this study that cross-linking of HA/CHI PEMs with GnP does not change the viscoelastic nature of these films, resulting in the poor adhesion observed. Whereas, in an alternate study, Schneider et al. (2007) [67] observed greatly improved chondrosarcoma cell adhesion towards EDC cross-linked HA/CHI multilayers, with HA-terminating films being preferred juxtaposed to CHI-terminated films. This increase in cell proliferation and adhesion comes with an increase in film stiffness (Young’s modulus ~15 kPa and 159 kPa for native and cross-linked films, respectively) and increased surface roughness.

Silva et al. (2015) [168] cross-linked ALG/CHI PEMs with GnP and reported an increase in film stability beyond pHs 3–9, outside of which the native PEMs were destabilised. The use of GnP allowed the films to retain their pH responsive properties while also increasing the range of pHs in which the films were stable. Moreover, using the same polymers and cross-linking agent, Silva et al. (2014) [132] demonstrated the increased stiffness of the PEMs compared to native films, with a reduction in film hydration. The increased stiffness resulted in greater L929 cell adherence, and fewer dead cells compared to native films, as well as greatly reduced aggregation. Similar results have been observed for that of myoblast differentiation upon HA/PLL films, which was shown to be strongly dependent upon the film stiffness [172]. Gribova et al. (2013) [176] demonstrated the enhanced migration and proliferation of C2C12 myoblasts, and the inhibition of myogenic differentiation upon stiffer poly L-glutamic/PLL PEMs, where softer PEMs allowed the differentiation to take place. Caridade et al. (2015) [177] reported similar results upon carbodiimide cross-linked ALG/CHI PEMs, in which myoblast proliferation was enhanced as function of cross-linking degree however, myoblast differentiation was also amplified; this, however, may be a result of different PEM hydration degrees or surface roughness, and perhaps the drastic difference in elastic moduli (>15 MPa and ~230 kPa for ALG/CHI and poly L-glutamic acid/PLL PEMs, respectively). Endothelial cells have also been shown to adhere and proliferate upon EDC cross-linked HA/PLL PEMs, with increasing tendency for the cells to undergo their endothelial-to-mesenchymal transition with increasing PEM stiffness [178], as well as leading to a long-term moderate increase in primary osteoblast proliferation on PLL/ALG and PLL/poly (galacturonic acid) [179] PEMs.

**Figure 13:**
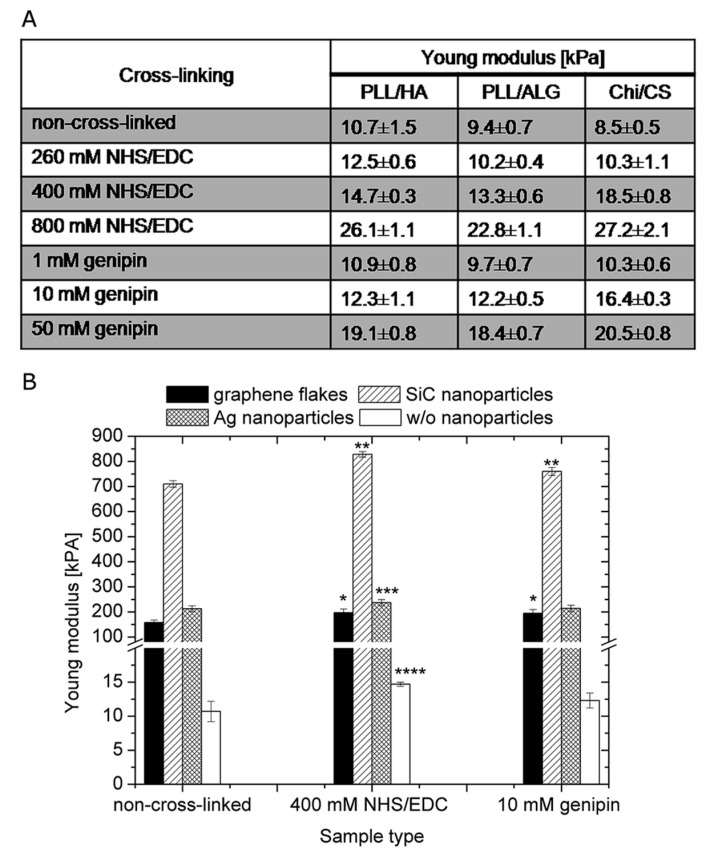
(**A**) The mechanical properties of non-cross-linked, NHS/EDC or genipin cross-linked PLL/HA, PLL/ALG and CHI/CS films. (**B**) The Young’s modulus of PLL/ HA films modified by graphene flakes, silicon carbide or silver nanoparticles, as well as films without nanoparticles is shown. Results obtained for non-cross- linked, 400 mM NHS/EDC and 10 mM genipin cross-linked samples are presented. * represents *p* < 0.05 vs. non-cross-linked sample with graphene flakes; ** represents *p* < 0.05 vs. non-cross-linked sample with SiC; *** represents *p* < 0.05 vs. non-cross-linked sample with Ag; **** represents *p* < 0.05 vs. non-cross-linked sample without nanoparticles. Figure taken from reference [183], ***C***opyright © 2020 Royal Society of Chemistry.

Indeed, the mechanical properties of films are generally improved upon cross-linking. Boudou et al. (2009) [180] reported a maximal elastic modulus (*E*) of 200 and 400 kPa for HA/CHI and HA/PLL PEMs cross-linked with EDC, very similar to that of Schneider et al. [174]. As a reference, the apparent Young’s modulus of non-cross-linked HA/PLL films ranges between 15 and 40 kPa [181]. To further increase the elastic modulus, the surface of the PEM can be modified; for instance, gold nanoparticles have been incorporated into HA/PLL PEMs, and it was found that the particles accumulated on the surface of the film and drastically increased the surface elastic modulus to a maximum of 1.3 MPa from 80 kPa [182]. Further surface modifications upon HA/PLL PEMs were performed by Mzyk et al. (2016) [183], in which, in combination with GnP or 400 mM EDC/NHS, silicon carbide nanoparticles were incorporated into the PEM, giving elastic moduli of 760 and 830 kPa, respectively (increased from ~10 kPa for native PEMs). Ag nanoparticles were also incorporated and showed an increase in the elastic modulus compared to native films, but showed no such drastic increase when compared to GnP cross-linked films (213 kPa and 214 kPa, respectively), but when used in combination with 400 mM EDC/NHS, the elastic modulus was raised to ~237 kPa. Further cross-linking modifications of HA/PLL and other PEMs can be seen in Figure 13. Other such large elastic moduli have been reported in CHI/ALG PEMs, with native CHI/ALG PEMs and EDC cross-linked films resulting in moduli of 3 MPa and >15 MPa, respectively [177]. Also, supplemental to these findings, Larkin et al. (2010) [184] reported the increase of young’s modulus from ~90 MPa to 310–480 MPa for glutaraldehyde cross-linked HA/CHI PEMs. From these reports, it can be said that effective cross-linking can give rise to substantial improvements in terms of the mechanical properties of PEM films.

It is important to note that mechanical properties of PEMs can also be tuned by their surface modification [106,182]. In this case, it is believed that internal dynamics of the polymers is not affected, but the exchange of polymers between the PEM stack and the surrounding media is usually suppressed by the capping layer [185,186]. In terms of film stability and structure, cross-linking may be the only reason the PEM can form; as aforementioned, Johnasson et al. (2005) [46] observed the instability of HA/COL PEMs at physiological pH, where cross-linking with carbodiimide chemistry together with NHS resulted in their stabilisation. Carbodiimide chemistry has also been employed within HA/PLL capsule systems to enhance stability [187] and reduce shrinkage [188], as well as within CMC/CHI PEMs to increase the porosity of the films, which was proven to increase the loading and release efficacy of macromolecular drugs [189].

### 3.3. Polymer Dynamics in Non-Enzymatic PEM Degradation

**Figure 14:**
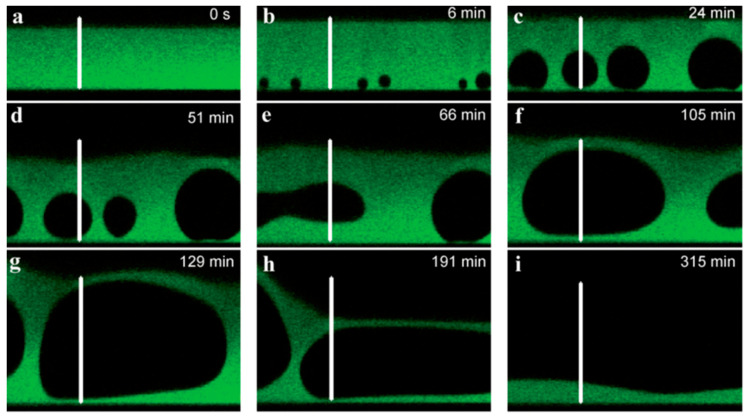
Vertical section of PEM observed by CLSM of a (PLL/HA)_50_/PLL^FITC^ film built in 0.15 M NaCl and swelled by immersion in solutions of increasing NaCl concentrations, up to 0.48 M, followed as a function of time. The scale bars represent 50 μm for (**a**,**b**,**c**), 60 μm for (**d**,**e**,**f**), and 80 μm for (**g**,**h**,**i**). Figure taken from reference [167], Copyright © 2020 Royal Society of Chemistry.

De-wetting of PEM films involves the retraction of the polymer film from the substrate surface, typically via the nucleation and formation of holes within the PEM, this can then lead to film rupture and dissolution. These holes generally form via heterogeneous nucleation, which arises from defects upon the substrate surface or can occur via residual stress being released into the system—from unfavoured polymer conformations, for instance [190,191]. One example is the ionic strength-induced de-wetting of HA/PLL PEMs; PEMs built up at 0.15 M NaCl placed in the presence of 0.48 M NaCl swell, but the film no longer remains homogenous, and holes begin to form within the PEM upon defects present. This is attributed to the continuously increasing diffusion of counter ions into the film, weakening the electrostatic cross-links between HA and PLL, and inducing an increase in osmotic pressure. These holes continue to grow and nucleate and may also coalesce; during this time HA and PLL chains within the PEM locally dissolve into the holes; there is a much higher osmotic pressure within the holes due to free polyelectrolytes bound to counter ions. Hence, overtime, the film is dramatically depleted in terms of its polymer content and an almost complete dissolution of the film is observed [167], as seen in Figure 14.

## 4. Summary and Perspectives

Like other aspects of LbL assembly, polymer dynamics in multilayer structures has been largely studied using synthetic films. Apparently, accumulated knowledge of the dynamics of polymers in such PEMs often can be applied to the biopolymer-based multilayers.

In this review, we sum up the main patterns of polymer dynamics established for synthetic films, i.e.,:

1. diffusion of polyelectrolyte molecules within PEMs is highly anisotropic, with the preferential diffusion parallel to the substrate [115].
2. the transition between linear and exponential film growth modes is closely correlated with the transition in polymer dynamics; at the transition point, film structure is changed from layered for linearly depositing films to highly intermixed for exponentially depositing LbLs [192].
3. polymer dynamics is influenced by the temperature, ionic strength and pH.

Many of these findings have been verified for biopolymer-based PEMs. Thus, it is important that polymer dynamics in biopolymer-based PEMs can be controlled by the same factors (temperature, ionic strength, pH, cross-linking) as synthetic PEMs. However, some aspects of biopolymer dynamics differ from the dynamics of synthetic analogues. For instance, the intrinsic polydispersity of biopolyelectrolytes and co-existence of multiple binding sites leads to the formation of several fractions of biopolymers within biopolymer-based PEMs. These fractions are characterized by different diffusivity that consequently reflects the formation, mechanical stability, and drug carrier and release properties of PEMs. Besides this, generally weak intermolecular interactions and high content of polymer-bound water results in the high dynamics of biopolymers.

Polymer diffusion in biopolymer-based PEMs is relatively fast and its investigation and quantification require higher spatial and temporal resolution of the methods. We expect that new analytical and theoretical simulation-based approaches for the investigation of molecular dynamics in thin films will emerge in future that will accelerate the research on biopolymer-based PEMs. New in vitro and in vivo studies of the interaction of biopolymer-based PEMs with cells and tissues are upcoming. We believe that deciphering of the mechanisms of dynamics of molecular signals in PEMs will be pivotal for fundamental understanding of PEM-cell interactions. In addition to this, the high promises of biopolymer LbL coatings of implants and substrates of sophisticated geometries will likely provoke the search of new methods for controllable LbL deposition; thus, we expect a breakthrough in microfluidic-based approaches. Another trend is the design of more complex hybrid ECM and ECM–cell interface mimicking materials. These composite and functional biopolymer-based PEMs will belong to the third generation of biomaterials where molecular dynamics of extracellular matrices is recapitulated with high spatiotemporal resolution, providing a powerful platform for active bio-mimicking coatings, for studying cell–ECM interactions, and for advanced tissue engineering.

## Figures and Tables

**Table 1:** Biopolymers and synthetic biodegradable polymers that are frequently used for the fabrication of PEMs. ALG—Alginic acid, CHI—Chitosan, CMC—Carboxymethylcellulose, COL—Collagen, CS—Chondroitin sulphate, DS—Dextran sulphate, FN—Fibronectin, GEL—Gelatin, HA—Hyaluronic acid, HS—Heparin sulphate, KF—Kafirin, PARG—Poly arginine, PEC—Pectic acid, PGA—Poly glutamic acid, PLL—Poly L-lysine, SF—Silk fibroin, TA—Tannic acid.

Polymer	Net Charge	Examples of Polymer Pair	Structure
1. Naturally occurring biopolymers			
Hyaluronic acid (HA)	−	PLL [42,43,44,45]; COL [46]; CHI [47,48]	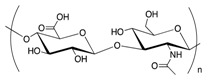
Collagen (COL)	+	HA [46,49]; HS [50,51,52]; HA, CS [53]; Lysozyme [54]; FN [55]	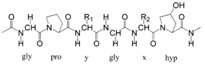
Alginic acid (ALG)	−	CHI [56,57,58,59]; PARG [60]; PLL [61]	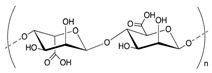
Gelatin (GEL)	+	TA [62]; SF [63]; PEC [64]; KF [65]	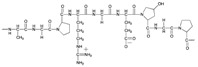
Chitosan (CHI)	+	HA [66,67] HS [68,69,70,71]; CS [72]; DS [73,74]; TA [75]; CMC [76,77,78]; Mucin [79]	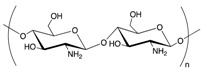
2. Synthetic biodegradable polymers			
Poly L-lysine (PLL)	+	CS [80,81,82]; HS [83,84]; HA [36,85]; Casein [86];	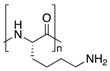
Poly arginine (PARG)	+	HA, GEL [87]; ALG, CS, HS, HA, PGA [88]; HA [89]; DS [90,91,92]; Casein [93]